# Hyper accuracy three-dimensional (HA3D™) technology for planning complex liver resections: a preliminary single center experience

**DOI:** 10.1007/s13304-022-01365-8

**Published:** 2022-08-25

**Authors:** Andrea Ruzzenente, Laura Alaimo, Simone Conci, Mario De Bellis, Andrea Marchese, Andrea Ciangherotti, Tommaso Campagnaro, Alfredo Guglielmi

**Affiliations:** Department of Surgery, Dentistry, Gynecology and Pediatrics, Division of General and Hepato-Biliary Surgery, University of Verona, P. le L.A. Scuro 10, 37134 Verona, Italy

**Keywords:** Three-dimensional visualization technology, Hepato-biliary surgery, Preoperative planning, Liver anatomy, Liver resection

## Abstract

Three-dimensional visualization technology (3DVT) has been recently introduced to achieve a precise preoperative planning of liver surgery. The aim of this observational study was to assess the accuracy of 3DVT for complex liver resections. 3DVT with hyper accuracy three-dimensional (HA3D™) technology was introduced at our institution on February 2020. Anatomical characteristics were collected from two-dimensional imaging (2DI) and 3DVT, while intraoperative and postoperative outcomes were recorded prospectively. A total of 62 patients were enrolled into the study. 3DVT was able to study tumor extension and liver anatomy, identifying at least one vascular variation in 37 patients (59.7%). Future remnant liver volume (FRLV) was measured using 2DI and 3DVT. The paired samples *t *test assessed positive correlation between the two methods (*p* < 0.001). At least one vessel was suspected to be invaded by the tumor in 8 (15.7%) 2DI cases vs 16 (31.4%) 3DVT cases, respectively. During surgery, vascular invasion was detected in 17 patients (33.3%). A total of 73 surgical procedures were proposed basing on 2DI, including 2 alternatives for 16 patients. After 3DVT, the previously planned procedure was changed in 15 cases (29.4%), due to the clearer information provided. A total of 51 patients (82%) underwent surgery. The most frequent procedure was right hepatectomy (33.3%), followed by left hepatectomy (23.5%) and left trisectionectomy (13.7%). Vascular resection and reconstruction were performed in 10 patients (19.6%) and portal vein was resected in more than half of these cases (66.7%). 3DVT leads to a more detailed and tailored approach to complex liver surgery, improving surgeons’ knowledge of liver anatomy and accuracy of liver resection.

**Graphical abstract:**

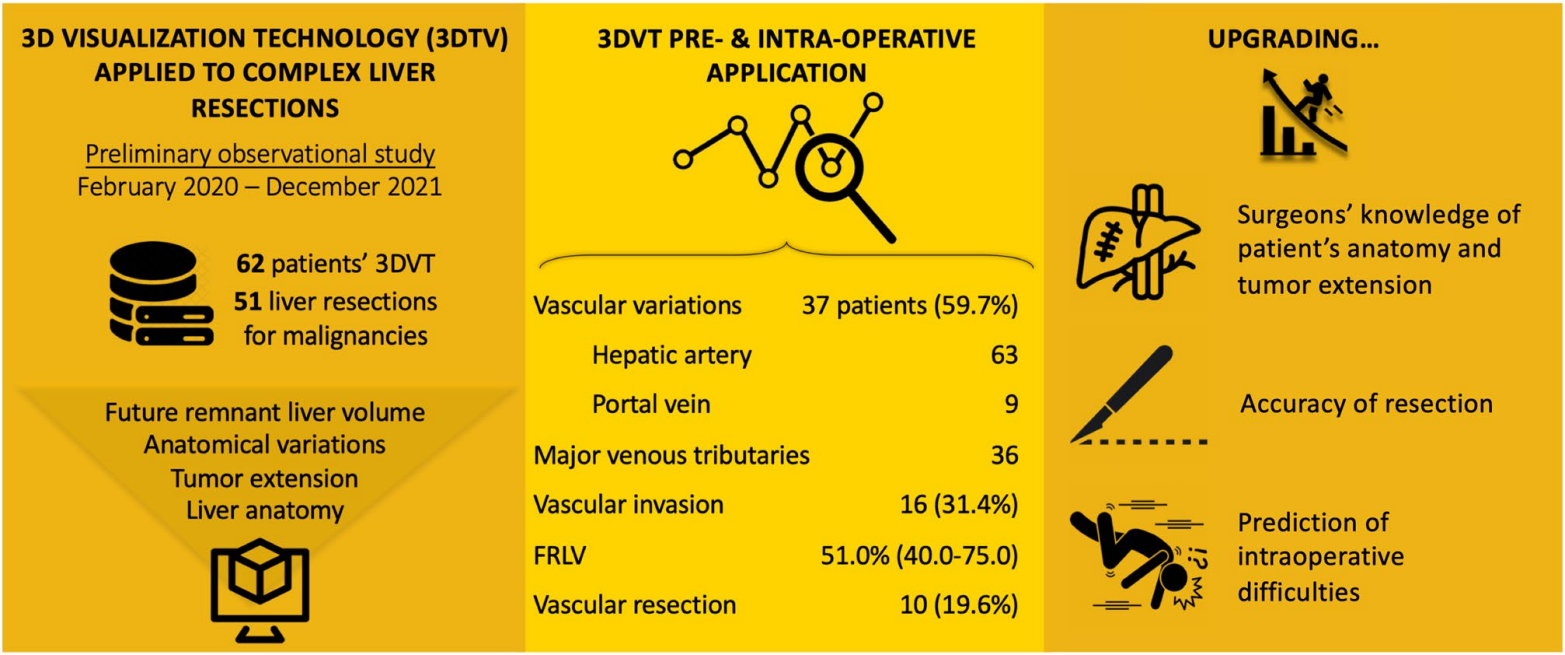

## Introduction

Three-dimensional (3D) technology has been recently introduced in clinical surgical practice. Specifically, in liver cancer surgery the precision of preoperative imaging is extremely important for diagnosis, for precise tumor localization, for estimation of resectability and to provide an accurate resection planning. The application of the most innovative technologies to liver surgery may allow to improve results, with increased accuracy and outcomes of liver resections. Among these, 3D visualization technology (3DVT) is an important field of research and it is gradually becoming an essential tool for hepatobiliary surgeons. Particularly, 3DVT is able to display, describe and explain morphology and spatial distribution of liver segments, biliary tree and blood vessels [1]. New additive manufacturing technologies with precise 3D print (3DP) of the liver and 3D intraoperative navigation (3DIN) systems are recent and innovative tools applied for visualization of 3D model. The application of the virtual environment during surgery using 3DIN allows the visualization in real time, improving the spatial orientation of surgeons and recall anatomical details during surgical procedure [2].

The utilization of 3DVT is useful in liver surgery for the complex segmental anatomy, the high frequency of individual variations and the complex relationship between tumors and main anatomical structures [2].

The application of 3DVT was proposed also to improve doctor-patient communication and to help patients in understanding the complexity of the disease and of planned surgical procedure [1].

Furthermore, 3DVT may facilitate the learning process of both medical students and residents, the precise spatial mapping of liver segments, the ability to locate the tumor. Tumor relationships with surrounding vascular and biliary structures are more easily identified by 3D technologies [3–5]. Consequently, a better comprehension of liver anatomy may improve surgical residents’ skills in both minimally invasive and open liver surgery [6].

In addition, the best knowledge of patient’s liver anatomy and tumor localization may improve the intraoperative confidence of the surgeon, leading to reduction of surgical time, number of complications, length of hospital stay and costs [7].

However, the utilization of 3DVT in liver surgery is recent, it needs validation from the scientific society and is still under evaluation in literature [8].

This study aimed to describe the results of a preliminary experience with 3DVT and 3DIN application to complex liver resections in a single center of hepatobiliary surgery.

## Methods

This prospective observational monocentric study was conducted at the Unit of Hepato-biliary surgery of the University of Verona from February 2020 to December 2021. The patients who were eligible for complex liver surgery were included, and 3DVT and 3DIN were applied for complex open and minimally invasive liver resections.

### Preoperative characteristics

The main demographic and clinical characteristics of patients were recorded during the preoperative medical examination, such as: gender, age, body mass index (BMI), comorbidities, liver function assessed by the evaluation of indocyanine green retention rate at 15 min (ICG R15), preoperative blood exams and preoperative diagnosis. In all cases future remnant liver volume (FRLV) of the planned resection was assessed using the computed tomography (CT) scans (two-dimensional imaging, 2DI) before the application of 3DVT. FRLV was expressed as a ratio of FRLV and total functioning liver volume (TLV). Liver volumetry was measured using a processing software from the 2DI. Liver volume calculation for 2DI was performed with standard procedure outlining liver, tumor and resection margins to calculate total liver volume, tumor volume and segmental planned remnant liver volumetry.

Portal vein embolization (PVE) was performed when the FRLV was lower than 40% of TLV, following the criteria previously published [9]. In these cases, the CT scans acquired 3–4 weeks after PVE were used for 3DVT. Liver volumetry after PVE was recorded to calculate the hypertrophy ratio using the following formula: hypertrophy ratio = 100 × (FRLV after PVE – FRLV before PVE)/FRLV before PVE [10].

The information collected from the 2DI reports was recorded, in particular: number of lesions, tumor size, tumor location, relationship between tumor and major vessels if available.

### 3D reconstruction of liver anatomy

Hyper accuracy 3D reconstructions with Hyper accuracy three-dimensional (HA3D™) technology of the images in DICOM format were processed by a dedicated software (MEDICS, Moncalieri, Turin, Italy). This technology was introduced at our institution in February 2020.

**Fig. 1:**
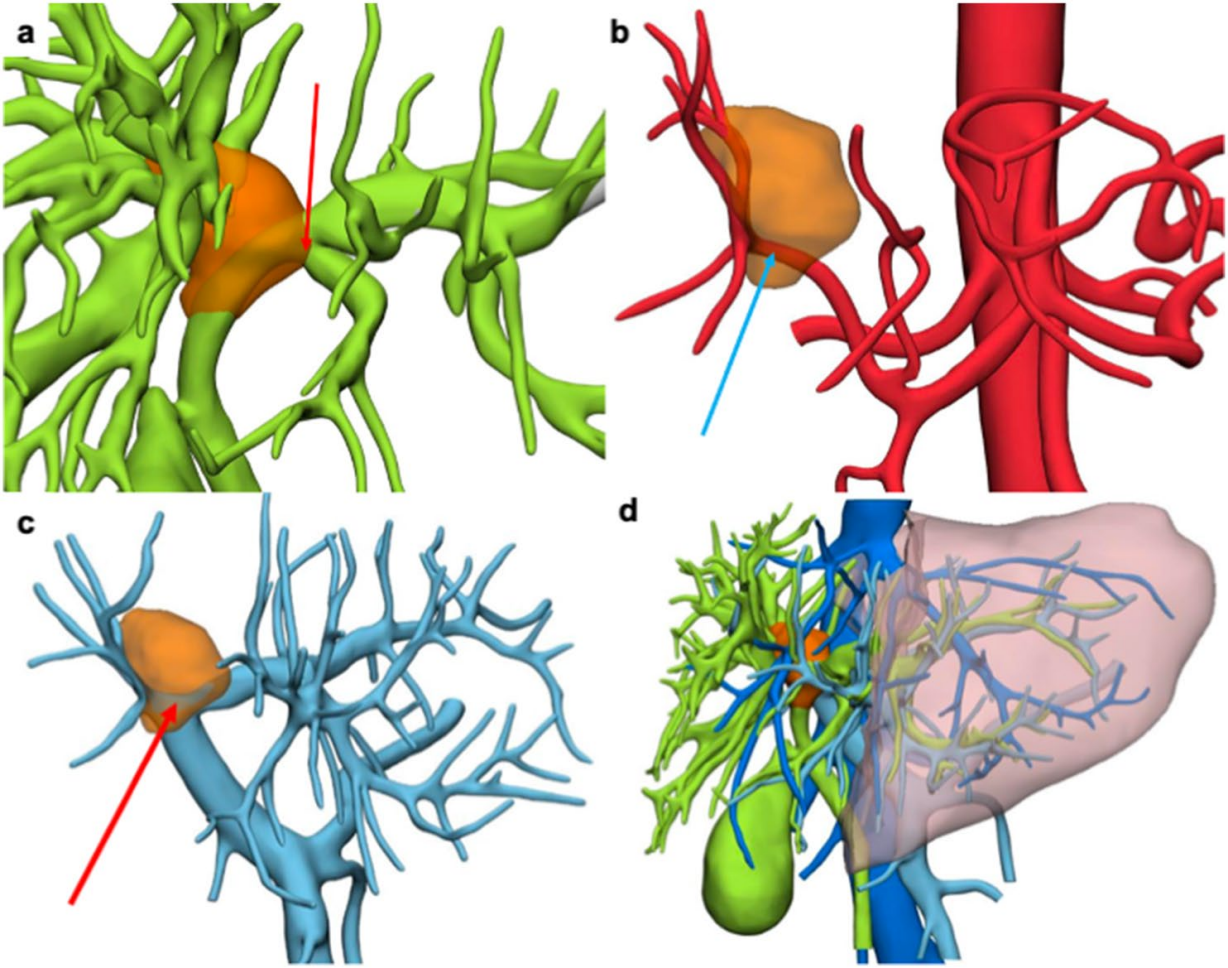
3D visualization technology of a case of peri-hilar cholangiocarcinoma. **a** Tumor extension (orange) to the right hepatic duct and left hepatic duct (segment 4 origin, narrow); **b** contact between the lesion (orange) and right hepatic artery (narrow); **c** contact between the lesion (orange) and right portal vein (narrow); **d** future remnant liver volumetry of right trisectionectomy

During the preoperative planning, complex cases of liver tumors were reconstructed to collect useful information on tumor location, anatomical variations of the main liver vessels and tumor involvement of biliary and vascular structures (Fig. 1). The definition of complex liver resection was based on the following characteristics: central tumor localization, suspicious vascular involvement, need for extended hepatectomy with vascular resection and reconstruction, biliary involvement by the tumor, and suspicious locally advanced disease.

**Fig. 2:**
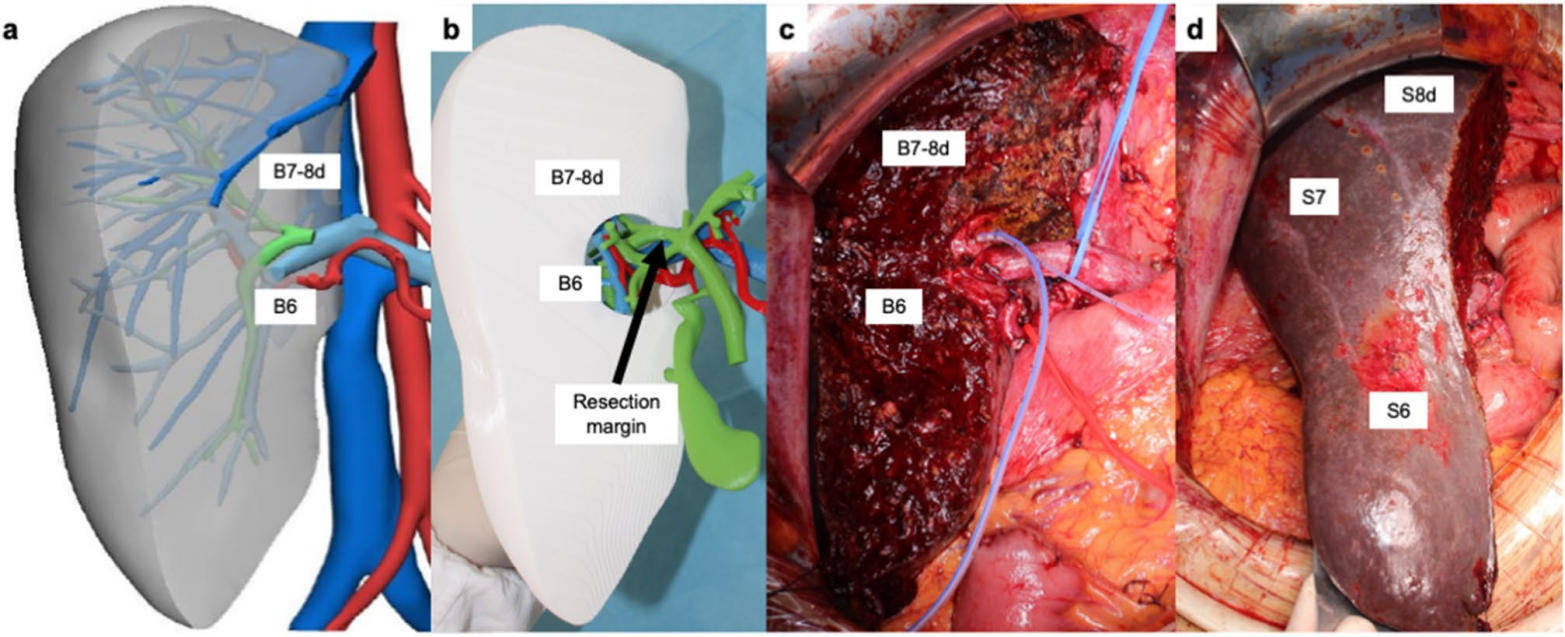
A case of left trisectionectomy preserving the dorsal biliary duct for segment 8 and the corresponding parenchyma for Bismuth-Corlette type IV peri-hilar cholangiocarcinoma. **a** 3D simulation of left trisectionectomy preserving the dorsal biliary duct for segment 8 (B8d) in addition to bile ducts for segment 6 (B6) and 7 (B7) and the corresponding parenchyma; **b** 3D printed model of the resection; **c**, **d** intraoperative image of remnant liver (segment 8 dorsal, S8d; segment 7, S7; segment 6, S6) and hilar structures after resection

The 3DVT was used during the preoperative planning to decide the most appropriate treatment in each case and intraoperatively to guide the surgeon during liver resection (Fig. 2).

The virtual simulation software allowed the surgeon to navigate each patient’s anatomy and hide the different structures of the liver as appropriate, to identify the exact location of the tumor and its proximity to blood vessels or biliary branches. Furthermore, virtual simulation of the resection and 3D liver volumetry helped the surgeon during the preoperative planning.

The 3D liver volumes were measured studying either portal perfusion and hepatic veins’ drainage, and included: TLV, tumor volume (TV), planned resection volume and FRLV (Fig. 1D).

### Blood vessels’ anatomical characteristics and variations

Vessels’ anatomical characteristics and variations were categorized according to the classifications previously published in literature [11–16].

The Michels’ classification of hepatic artery anatomy described 10 types of variations: type I, normal pattern; type II, replaced left hepatic artery (LHA) from left gastric artery (LGA); type III, replaced right hepatic artery (RHA) from superior mesenteric artery (SMA); type IV, replaced LHA and RHA (types II + III); type V, accessory LHA from LGA; type VI, accessory RHA from SMA; type VII, accessory LHA and RHA (types V + VI); type VIII, accessory LHA and replaced RHA (types V + III); IX type, replaced common hepatic artery from SMA; type X, replaced common hepatic artery from LGA [13].

Portal vein anatomy according to the Couinaud’s classification may vary into 5 types: type I, normal pattern; type II or portal vein trifurcation, the main portal vein (MPV) is divided into right anterior (RAPV), right posterior (RPPV) and left portal veins (LPV); type III, the RPPV arises directly from MPV as its first branch, at the lower part of hepatic hilum, and LPV is the terminal branch, arising after the origin of the RAPV; type IV or trifurcation of right portal vein (RPV), segment 7 branch is the first branch of RPV; type V, segment 6 branch arises early as first branch of RPV [14, 16].

The hepatic veins’ drainage was mapped in detail by Tani et al., who classified the major tributaries as following: tributaries of left hepatic vein, such as left superficial vein and umbilical fissure vein; tributaries of middle hepatic vein, such as superior or inferior vein for segment 4 (V4sup and V4inf, respectively), ventral or intermediate vein for segment 8 (V8v and V8i, respectively), and veins for segment 5; tributaries of right hepatic vein, such as right superficial vein and dorsal vein for segment 8; accessory veins, such as inferior right hepatic vein (IRHV) and middle right hepatic vein [15, 17]. Most of those tributaries are usually too thin to be clearly observed at the CT scans, hence in this study only the veins with a diameter greater than 3 mm were considered major tributaries and recorded [17].

Finally, the relationship between the tumor and the major vessels was assessed by the 2DI, 3DVT and intraoperatively, basing on the characteristics of vessels’ shape and diameter [1, 12, 18].

### Intraoperative and postoperative outcomes

The surgical procedures were performed by four expert hepatobiliary surgeons (AG, AR, TC, SC).

The intraoperative data were collected from surgical reports, including type of liver resection, tumor-vessels’ relationship and vascular resections. The nomenclature of hepatectomies was defined according to the Brisbane 2000 terminology of liver resections [19].

**Fig. 3:**
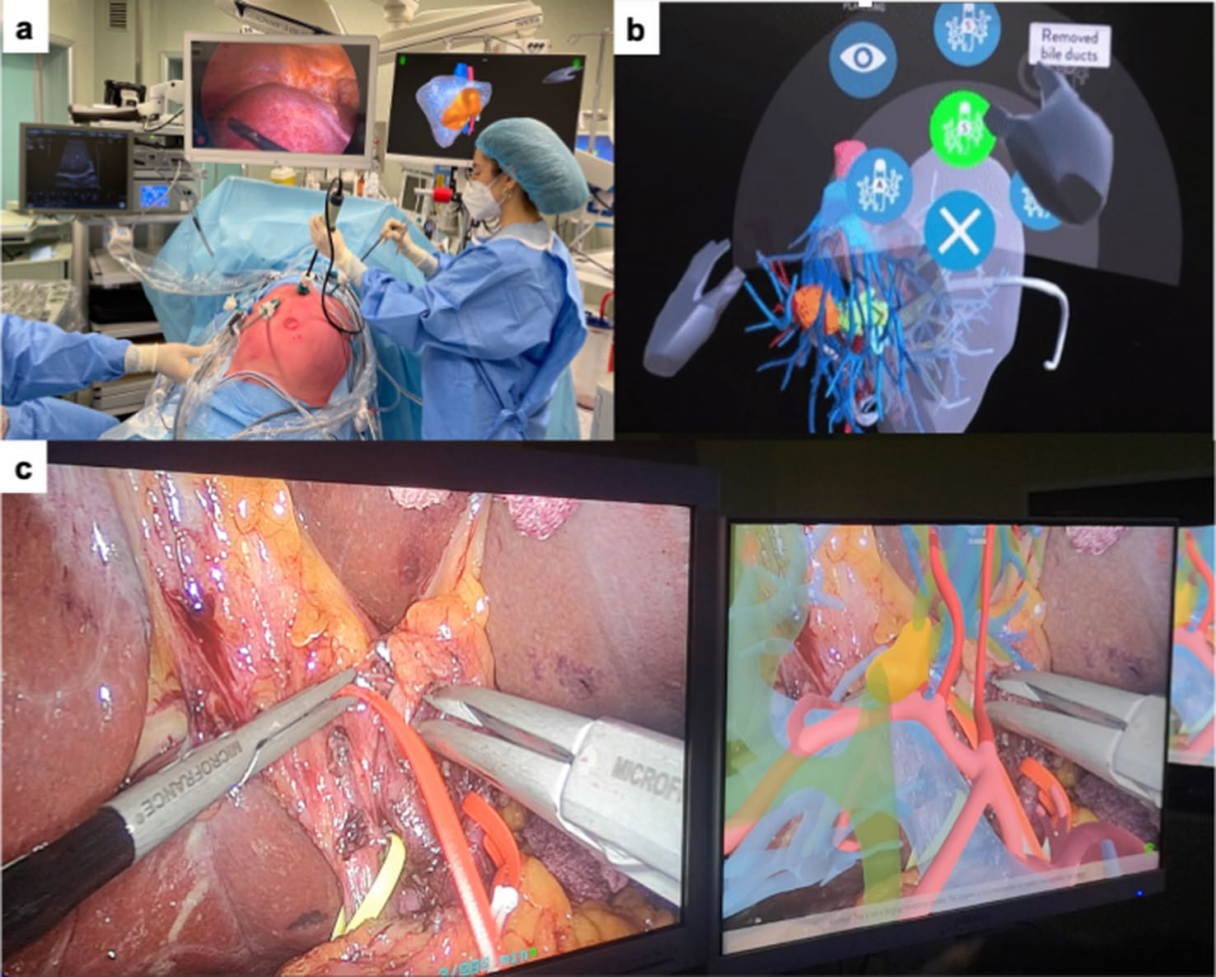
Intraoperative cognitive navigation system (ICON). **a** Setting of the operative room for laparoscopic liver resection; **b** ICON touchless manipulation of the 3D model; **c** intraoperative surgical field and superimposing of the 3D model

The intraoperative navigation device ICON (Intraoperative COgnitive Navigation system, MEDICS, Moncalieri, Turin, Italy), was applied for intraoperative 3DVT evaluation (Fig. 3). The ICON device allowed the surgeon to manipulate 3DVT using a touchless system during surgery, to establish the tumor extension, identify the principal vessels, help achieving the oncological radicality and decreasing the risk of complications. The postoperative outcomes were recorded prospectively, including postoperative complications, length of hospital stay (LOS) and death within 30 days from surgery. The comprehensive complication index was calculated to assess the global morbidity of each patient [20].

### Statistical analysis

The software SPSS (Version 25.0, Chicago, IL) was used to carry out the statistical analysis. The continuous variables were expressed as median and the corresponding interquartile range (IQR) or mean ± standard deviation (SD). The categorical variables were expressed as counts and percentages. The paired samples t-test was used to analyze the correlation between 2DI and 3DVT measurements of FRLV. The Bland–Altman plot was applied to assess the agreement between the 2DI and the 3DVT, as two different methods of FRLV measurement. Then, the regression linear analysis was performed to reveal the grade of agreement. The *p* value ≤ 0.05 established the statistical significancy.

## Results

A total of 62 cases were reconstructed by 3DVT and enrolled into the study. The clinical data of each patient were collected in a prospective database from February 2020 to December 2021. During this period, 51 patients (82%) underwent surgery and the postoperative short-term outcomes were recorded.

### Preoperative characteristics

The main preoperative characteristics are reported in Table 1. Among the 62 cases included into the study, the 61.3% (*n* = 38) were men and the median age of the patients was 69.0 years old (IQR, 60.5–76.5). The median BMI was 25.0 kg/m^2^ (IQR, 22.2–27.8). More than half of the patients had comorbidities increasing the operative risks, according to the American Society of Anesthesiologists’ physical status classification system (ASA score ≥ 3, 54.7%). The most frequent diagnosis was peri-hilar cholangiocarcinoma (PHCC) (*n* = 30, 48.4%), followed by intrahepatic cholangiocarcinoma (iCCC) (*n* = 12, 19.3%), hepatocellular carcinoma (HCC) (*n* = 10, 16.1%), gallbladder cancer (GBC), colorectal liver metastases (CRLM) (both *n* = 4, 6.4%) and primary sclerosing cholangitis (PSC) (*n* = 2, 3.2%). The median number of lesions detected was 1 (range, 1–7) and the median tumor size was 30.0 mm (IQR, 19.5–72.5). PBD was necessary in the 40.3% of the patients (*n* = 25) to relieve jaundice. The most frequent method of PBD was PTBD (*n* = 19). The ICG R15 assessment of liver function showed a mean value of 9.8% (± 7.7). PVE was performed in the 16.1% of patients (*n* = 10), being the FRLV lower than 40% of TLV. The hypertrophy ratio after PVE was 51.0% (IQR, 35.8–129.0) and the median time from PVE to surgery was 36 days (IQR, 33.5–49.7). The median time from 2DI and 3DVT was 24 days (IQR, 11–41). While the median time from 3DVT and surgery was 8 days (IQR, 2–25).

**Table 1:** Patients’ preoperative characteristics

Variables	*n* = 62
Gender (male), *n (%)*	38 (61.3)
Age (year), *median (IQR)*	69.0 (60.5–76.5)
BMI (kg/m^2^), *median (IQR)*	25.0 (22.2–27.8)
ASA ≥ 3, *n (%)*	29 (54.7)
ICG R15, *mean (*± *SD)*	9.8 (± 7.7)
Total Bilirubin (mg/dL), *median (IQR)*	0.8 (0.6–1.8)
INR, *median (IQR)*	1.05 (1.0–1.1)
Albumin (g/L), *median (IQR)*	37.9 (34.0–42.2)
Platelet’s count (cell/mm^3^), *median (IQR)*	247,000 (190,500–323,500)
Hemoglobin (g/dL), *median (IQR)*	12.8 (11.2–13.8)
Preoperative diagnosis, *n (%)*	
PHCC	30 (48.4)
iCCC	12 (19.3)
HCC	10 (16.1)
GBC	4 (6.4)
CRLM	4 (6.4)
PSC	2 (3.2)
Number of lesions, *median (range)*	1 (1–7)
Tumor size, *median (IQR)*	30.0 mm (19.5–72.5)
PBD, *n (%)*	25 (40.3)
EBD	6 (9,7)
PTBD	19 (30.6)
PVE, *n (%)*	10 (16.1)
Hypertrophy ratio after PVE, *median (IQR)*	51.0% (35.8–129.0)
Time from PVE to surgery, *median (IQR)*	36 days (33.5–49.7)
2DI with PBD, *n (%)*	7 (11.3)
Time from 2DI to 3DVT, *median (IQR)*	24 days (11–41)
Time from 3DVT to surgery, *median (IQR)*	8 days (2–25)

BMI: body mass index. ASA: American Society of Anesthesiologists’ physical status classification system. ICG R15: indocyanine green retention rate at 15 min. INR: international normalized ratio. PHCC: peri-hilar cholangiocarcinoma. HCC: hepatocellular carcinoma. GBC: gallbladder cancer. iCCC: intrahepatic cholangiocarcinoma. CRLM: colorectal liver metastases. PSC: primary sclerosing cholangitis. PBD: preoperative biliary drainage. EBD: endoscopic biliary drainage. PTBD: percutaneous transhepatic biliary drainage. PVE: portal vein embolization. 2DI with PBD: 2D imaging performed in presence of PBD

### 3D visualization technology description of vascular anatomy

Descriptive data of the 3DVT are reported in Table 2. The 3DVT identified at least one vascular variation in 37 patients (59.7%) and 21 of them (56.7%) had more than one variation. In particular, the 3DVT assessed 63 variations of the hepatic artery anatomy. According to Michels classification the most frequent variations were type III and V (both 19.0%, *n* = 12), followed by type VI (4.8%, *n* = 3), type IX (3.2%, *n* = 2). A total of 33 (52.4%) variations were not described by the classification and the most frequent of them was the presence of LHA arising from celiac trunk (*n* = 7, 11.1%) and accessory LHA from proper hepatic artery (*n* = 5, 7.9%).

**Table 2:** 3D visualization technology description of vascular anatomy

Variables	*n* = 62
Vascular anatomical variations, *n patients (%)*	37 (59.7%)
Hepatic artery, *n (%)*	63
Type II	1 (1.6)
Type III	12 (19.0)
Type V	12 (19.0)
Type VI	3 (4.8)
Type IX	2 (3.2)
Not classified types	33 (52.4)
Portal vein, *n (%)*	9
Type I	2 (22.2)
Type II	3 (33.3)
Type III	1 (11.1)
Type IV	1 (11.1)
Not classified types	2 (22.2)
Major hepatic venous tributaries, *n (%)*	36
IRHV	30 (83.3)
V4inf + V8v	1 (2.8)
Presence of shunts	5 (13.9)

Michels classification of hepatic artery variations: type II, left hepatic artery (LHA) from left gastric artery; type III, right hepatic artery (RHA) from superior mesenteric artery (SMA); type V, accessory LHA from left gastric artery; type VI, accessory RHA from SMA; type IX, common hepatic artery from SMA. Major hepatic venous tributaries: defined by a diameter ≥ 3 mm; Portal vein’s variations: type I, main portal vein (PV) divided into left, right anterior and right posterior branches; type II, main PV sends out the right posterior branch and then divides into left and right anterior branches; type III, right PV is divided horizontally into anterior and posterior branches; type IV, horizontal part of the left PV is missing and the left PV is from the right anterior branch. IRHV, presence of inferior right hepatic vein draining the Sg6 into inferior vena cava; V4inf + V8v, inferior vein for Sg4 and ventral vein for Sg8 tributaries of the middle hepatic vein

A total of 9 patients (14.5%) had variation of portal vein anatomy, in particular: type I, 2 patients (22.2%); type II, 3 patients (33.3%); type III, 1 patient (11.1%) and type IV, 1 patient (11.1%), 2 patients (22.2%) had non-classified variations.

Eventually, major hepatic venous tributaries (≥ 3 mm in diameter) were detected by 3DVT in a total of 32 patients (51.6%) and 4 (12.5%) patients of them had more than one major tributary. In particular, a major inferior right hepatic vein was assessed in 30 cases (83.3%), only 1 patient had both major inferior vein for segment 4 and major ventral vein for segment 8. Furthermore, the presence of intrahepatic venous shunts was evident in 5 cases (13.9%).

The 2DI identified at least one vascular variation in 31 cases (50.0% of patients), while 3DVT described vascular variations in 37 cases (59.7% of patients). The same variations were found by the two methods. The 2DI was able to identify vascular variations in most cases with an agreement between the two modalities of 83.7%.

### Surgical planning

The data of surgical planning are reported in Table 3. According to liver volumetry assessed using the CT scans, the median FRLV of the resection planned was 51.0% (IQR, 38.7–73.8), while the FRLV of the alternative resection was 39.4% (29.2–56.4). The median FRLV of the first resection proposed was 51.0% (IQR, 40.0–75.0) and it was 43.0% (IQR, 31.5–58.5) for the alternative planned resection.

**Table 3:** Surgical planning

Variables	*n* = 62
FRLV 1 (%), *median (IQR)*	
2DI	52.0 (38.7–73.8)
3DVT	51.0 (40.0–75.0)
FRLV 2 (%), *median (IQR)*	
2DI	39.4 (29.2–56.4)
3DVT	43.0 (31.5–58.5)
Planned resection modifications, *n* (%)	15 (29.4)
Resectable⇨ Unresectable	4 (7.8)
Extended hepatectomy⇨ Major	4 (7.8)
Major hepatectomy⇨ Extended	2 (3.9)
Major resection⇨ Minor	1 (2.0)
Anatomical resection⇨ Non-anatomical	1 (2.0)

FRLV: future remnant liver volume, 1 and 2 were the two alternatives proposed

At the paired samples t-test comparing the measurements of the FRLV assessed using 2DI and 3DVT, the two methods were significantly positively correlated (*r* = 0.781, *p* < 0.001).

**Fig. 4:**
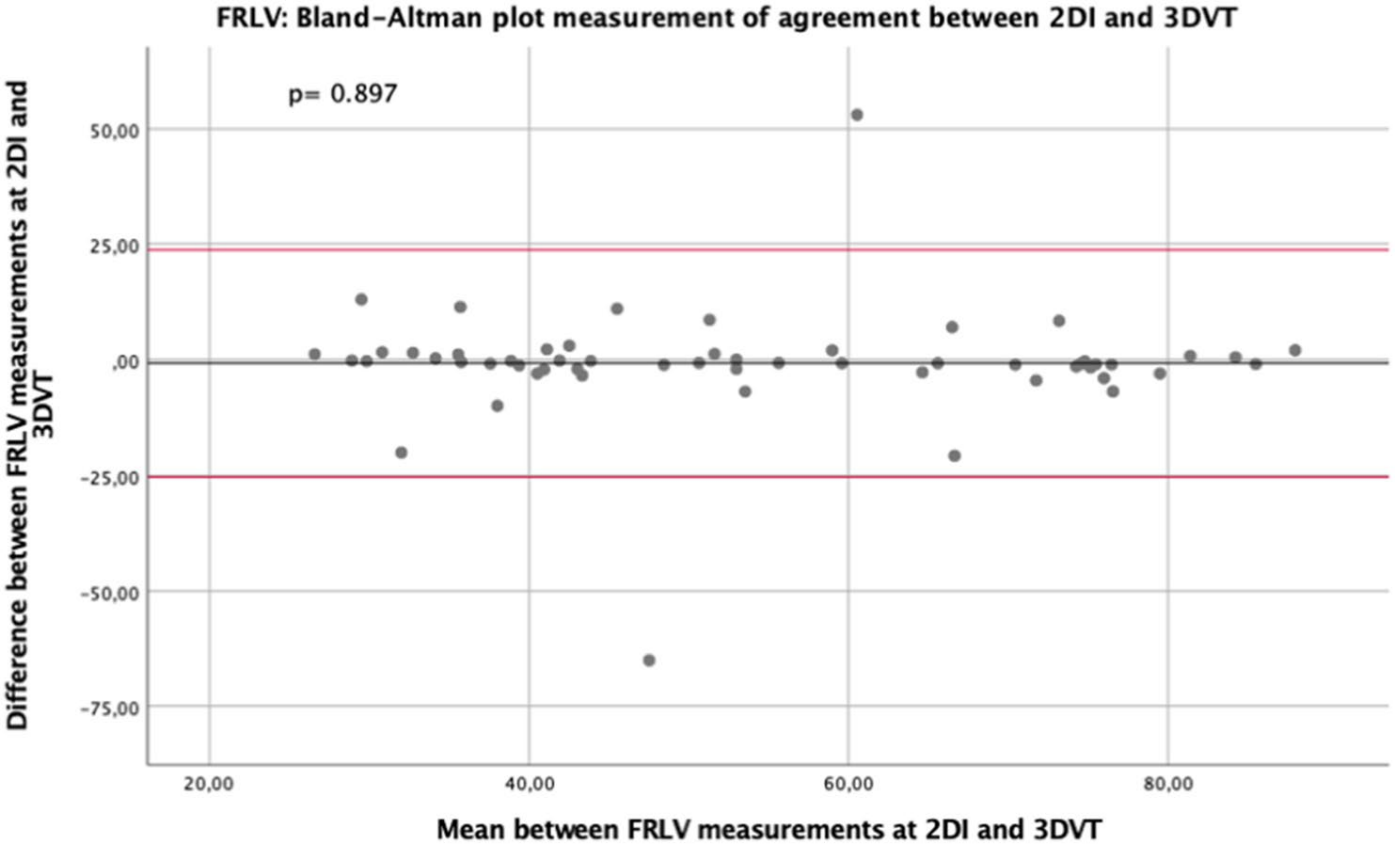
Bland–Altman plot assessing agreement between 2DI and 3DVT measurement of future remnant liver volume

Furthermore, the Bland–Altman plot was performed to compare the use of 2DI and 3DVT for estimation of FRLV (Fig. 4) and they did not achieve the statistically significance (*p* = 0.897), confirming the good agreement between the two methods of FRLV estimation.

After a multidisciplinary evaluation of each case, a total of 71 surgical procedures were proposed, with 2 different alternatives in 13 cases. However, after the 3DVT evaluation, the comprehension of tumor location and extension was more precise and the previously procedure planned was changed in 15 cases (29.4%). In particular, in 4 cases (7.8%) the disease was locally advanced and patients were candidates for neoadjuvant therapy, while in 4 cases (7.8%) the resection was modified from extended hepatectomy to major, in 2 cases (3.9%) it was modified from major hepatectomy to extended, in 1 case (2.0%) from major to minor resection and in 1 case (2.0%) from the anatomical resection into a non-anatomical one.

The preoperative evaluation of tumor-vessels’ relationship revealed a higher number of suspected vascular invasions at the 3DVT in comparison with 2DI (31.4% vs 15.7%, respectively). This result was similar to the intraoperative evidence, where the surgeon found at least one vessel invaded in 17 patients (33.3%). Hence, while 2DI had a greater specificity than 3DVT [91.2% (95% CI, 76.3–98.1) vs 80.6% (95% CI, 64.0–91.8), respectively], 3DVT had a greater sensitivity than 2DI [52.9% (95% CI, 27.8–77.0) vs 29.4% (95% CI, 10.3–56.0), respectively].

### Intraoperative and postoperative outcomes

The intraoperative data and the postoperative outcomes are reported in Table 4. A total of 51 (82.3%) patients underwent liver resection after 3DVT application. The most frequent surgical procedure was right hepatectomy (33.3%, *n* = 17), followed by left hepatectomy (23.5%, *n* = 12), minor resections (21.6%, *n* = 11), left trisectionectomy (13.7%, *n* = 7), right trisectionectomy and mesohepatectomy (both 3.9%, *n* = 2). Vascular resection and reconstruction were performed in 10 patients (19.6%), PV was the most frequent resected vessel (66.7%, *n* = 8).

**Table 4:** Surgical, histological and postoperative outcomes

Variables	*n* = 51
Type of resection, *n (%)*	51 (82.3)
Right hepatectomy	17 (33.3)
Left hepatectomy	12 (23.5)
Minor resections	11 (21.6)
Left trisectionectomy	7 (13.7)
Right trisectionectomy	2 (3.9)
Mesohepatectomy	2 (3.9)
Vascular resection and reconstruction, *n (%)*	10 (19.6)
Portal vein	8 (66.7)
Hepatic artery	1 (8.3)
IVC, partial lateral resection	1 (8.3)
Blood vessels histological involvement, *n (%)*	26 (51.0)
Right hepatic artery	2 (7.7)
Right portal vein	12 (46.1)
Left portal vein	5 (19.2)
Right hepatic vein	4 (15.4)
Middle hepatic vein	1 (3.8)
IVC	2 (7.7)
Tumor size, *median (IQR)*	36.5 mm (20.0–72.5)
R0 resection, *n (%)*	40 (78.4)
Global morbidity (CCI), *median (IQR)*	29.6 (20.9–40.8)
LOS (days), *median (IQR)*	10 (7–17)
30-day mortality, *n (%)*	3 (5.9)

IVC: inferior vena cava. R0 resection: microscopically negative margins of resection. CCI: comprehensive complication index. LOS: length of hospital stay

The median tumor size was 36.5 mm (IQR, 20.0–72.5) and the paired samples *t *test confirmed good correlation of tumor size measurement among histological examination and both 3DVT (*r* = 0.936, *p* < 0.001) and 2DI (*r* = 0.862, *p* < 0.001).

The median global postoperative morbidity assessed by comprehensive complication index (CCI) was 29.6 (IQR, 20.9–40.8). The median length of hospital stay (LOS) was 10 days (IQR, 7–17). A total of 3 patients died within 30 days from surgery (5.9%).

## Discussion

In the field of liver cancer surgery, the accurate preoperative radiological findings allow to predict technical resectability. The application of the modern 3D technology may add further details to 2DI and improve surgical outcomes. In particular, the precise description of tumor location, tumor extension, pattern of tumor borders and its relationships with surrounding structures may greatly influence resectability and type of surgical procedure [21–23].

In literature, a good correspondence between 3DVT and surgical specimen was clearly demonstrated. Yang et al. compared 3DVT, 3DP and 2DI of patients undergoing liver resection for oncological disease. The Authors found a significant improvement in the accuracy of tumor location and preoperative planning of resection using 3D technology, in particular 3DP [6]. Lopez et al. analyzed the accuracy and usefulness of 3D printed models (3DP) applied to liver surgery, learning curve and doctor–patient communication. In particular, the Authors reported a good agreement between 3D technologies and 2DI in terms of measurement of vessels’ and bile ducts’ calibers and their distances from tumor. Also, resection margins predicted by the 3DP were accurate and comparable with the surgical specimen [3]. Among methods displaying 3DVT, we applied an innovative device for intraoperative navigation that allowed the surgeon to manipulate 3DVT during surgery allowing a more precise resection.

Liver anatomy is characterized by high variability among the individuals. A precise definition of liver segments’ portal and arterial supply, and venous drainage may allow to plan more accurate anatomic liver resections. In addition to the information provided by the 2DI, the 3DVT ability to reconstruct and intuitively display vessels may improve significantly the preoperative planning based on the morphological features of blood vessels [17, 24].

The 3DVT provided a great number of detailed information on the vessels’ anomalies, their course and relationship with the tumor. We identified 63 variations of hepatic artery, in 37 patients (59.7%), 21 of them (56.7%) with multiple variations. Evidence of major venous variants was described by the 3DVT in 32 patients (51.6%).

The 3DVT demonstrated to be more accurate than 2DI in the identification of liver lesions, which are displayed in the different spatial projections, hence it may reduce some subjective errors during preoperative planning and evaluation of vascular invasion [24]. Zhang et al. published their experience with the 3DVT applied to diagnosis of portal vein invasion during the preoperative planning of PHCC. The Authors reported an increased sensitivity, specificity and overall accuracy of 3DVT visualization in comparison with the standard 2DI evaluation [25]. In the present study the evaluation of tumor involvement of blood vessels was performed by expert radiologists and surgeons, who found a better agreement between 3DVT and surgery than 2DI. We observed tumor invasion of at least one vessel in 8 (15.7%) patients’ 2DI, in 16 (31.4%) patients’ 3DVT and 17 (33.3%) patients’ intraoperative findings. The precision of representing tumor–vessels’ relationships using 3DVT may reduce unnecessary vascular dissections or resections, decreasing considerably the risk of complications, such as bleeding or devascularization of biliary tree [8].

The accuracy of 3DVT in displaying liver and vessels anatomy, tumor relationship with the surrounding structures and measurement of FRLV allows the surgeons to plan a more accurate liver resection and be more confident during surgery, due to the previous detailed comprehension of patients’ anatomy [7]. Witowski et al. underlined the relevant role of 3DVT during the preoperative planning influencing the resection proposal [26]. In our experience the type of resection planned after the evaluation of 2DI was changed by the surgeon after the 3DVT in 29.4% of the cases (*n* = 15) because of the clearer information provided. In literature, the indications for application of 3DVT to liver surgery have not been well established. It increases the costs, and the majority of the studies focus on the technical aspects instead of the quantitative statistical and clinical results [5, 27]. Nowadays, the most reasonable indication seems to apply these technologies to complex cases with high risk of complications [23].

The results of this preliminary study have some limitations that should be addressed.

Firstly, the sample size is small because of the recent introduction of the 3D technology at our institution. Secondly, in this preliminary study we included different types of liver tumor. A study regarding the 3DVT application to a single complex disease is planned to achieve more precise and focused results and identify the 3DVT added value on the postoperative short- and long-term outcomes.

Thirdly, the evaluation of tumor-vessels’ involvement was subjective, but performed by expert radiologists and surgeons. However, in future the application and validation of a specific 3DVT artificial intelligence algorithm could increase the precision of 3D technology.

## Conclusions

In conclusion, the application of 3D technology to liver surgery is promising, because it may improve surgeons’ perception of liver vascular and biliary anatomy, tumor extension and relationship with main liver structures. All of these aspects may enhance the accuracy of preoperative resection planning, prediction of intraoperative complications and of postoperative outcomes. Hence, the application of 3DVT to complex liver resections is certainly promising, although it needs larger population and validation studies focused on the clinical outcomes.
